# The transcriptional regulator Lrp activates the expression of genes involved in the biosynthesis of tilimycin and tilivalline enterotoxins in *Klebsiella oxytoca*

**DOI:** 10.1128/msphere.00780-24

**Published:** 2024-12-17

**Authors:** Miguel A. De la Cruz, Hilda A. Valdez-Salazar, Diana Rodríguez-Valverde, Santa Mejia-Ventura, Nayely Robles-Leyva, Tania Siqueiros-Cendón, Quintín Rascón-Cruz, Nancy León-Montes, Jorge Soria-Bustos, Fernando Chimal-Cázares, Roberto Rosales-Reyes, María L. Cedillo, Jorge A. Yañez-Santos, J. Antonio Ibarra, Javier Torres, Jorge A. Girón, James G. Fox, Miguel A. Ares

**Affiliations:** 1 Unidad de Investigación Médica en Enfermedades Infecciosas y Parasitarias, Centro Médico Nacional Siglo XXI, Instituto Mexicano del Seguro Social, Mexico City, Mexico; 2 Facultad de Medicina, Benemérita Universidad Autónoma de Puebla, Puebla, Mexico; 3 Centro de Detección Biomolecular, Benemérita Universidad Autónoma de Puebla, Puebla, Mexico; 4 Facultad de Ciencias Químicas, Universidad Autónoma de Chihuahua, Chihuahua, Mexico; 5 Instituto de Ciencias de la Salud, Universidad Autónoma del Estado de Hidalgo, Tulancingo, Hidalgo, Mexico; 6 Unidad de Medicina Experimental, Facultad de Medicina, Universidad Autónoma de México, Mexico City, Mexico; 7 Departamento de Microbiología, Escuela Nacional de Ciencias Biológicas, Instituto Politécnico Nacional, Mexico City, Mexico; 8 Division of Comparative Medicine, Massachusetts Institute of Technology, Cambridge, Massachusetts, USA; University of Michigan-Ann Arbor, Ann Arbor, Michigan, USA

**Keywords:** *lrp*, citotoxicity, *aroX*, *npsA*, *Klebsiella oxytoca*

## Abstract

The toxigenic *Klebsiella oxytoca* strains secrete tilymicin and tilivalline enterotoxins, which cause antibiotic-associated hemorrhagic colitis. Both enterotoxins are non-ribosomal peptides synthesized by enzymes encoded in two divergent operons clustered in a pathogenicity island. The transcriptional regulator Lrp (*l*eucine-responsive *r*egulatory *p*rotein) controls the expression of several bacterial genes involved in virulence. In this work, we have uncovered novel findings that have significant implications. We determined the transcriptional expression of *aroX* and *npsA*, the first genes of each tilimycin (TM)/tilivalline (TV) biosynthetic operon in *K. oxytoca* MIT 09-7231 wild-type and its derivatives Δ*lrp* mutant and complemented strains. Our results suggest that Lrp directly activates the transcription of both *aroX* and *npsA* genes by binding to the intergenic regulatory region in a leucine-dependent manner. Furthermore, the lack of Lrp significantly diminished the cytotoxicity of *K. oxytoca* on HeLa cells due to reduced production of TM and TV. Altogether, our data present a new perspective on the role of Lrp as a regulator in cytotoxin-producing *K. oxytoca* strains and how it controls the expression of genes involved in the biosynthesis of their main virulence factors.

**IMPORTANCE:**

Tilimycin (TM) and tilivalline (TV) are enterotoxins that are a hallmark for the cytotoxin-producing *Klebsiella oxytoca* strains, which cause antibiotic-associated hemorrhagic colitis. The biosynthesis of TM and TV is driven by enzymes encoded by the *aroX*- and NRPS-operons. In this study, we discovered that the transcriptional regulator Lrp plays a crucial role in activating the expression of the *aroX*- and NRPS-operons, thereby initiating TM and TV biosynthesis. Our results underscore a molecular mechanism by which TM and TV production by toxigenic *K. oxytoca* strains is regulated and shed further light on developing strategies to prevent the intestinal illness caused by this enteric pathogen.

## INTRODUCTION

*Klebsiella oxytoca* is an opportunistic Gram-negative bacterium that resides in the colon and, after a β-lactam antibiotic treatment, utilizes its constitutively expressed β-lactamase to survive. In contrast, other commensal bacteria are eliminated (1). Pathobiont *K. oxytoca* is the causative agent of antibiotic-associated hemorrhagic colitis (AAHC) (2). The pathobiont *K. oxytoca* initiates dysbiosis in the absence of a competitive microbial community (3). This condition, dysbiosis, involves a loss of commensals and an expansion of pathobionts (4, 5). The subsequent proliferation of *K. oxytoca* in the colon results in the overproduction of tilimycin (TM) and tilivalline (TV) enterotoxins. *K. oxytoca* synthesizes TM and TV via non-ribosomal peptide synthases encoded by the *aroX*- and NRPS-operons, which are part of a pathogenicity island (PAI) (6–8).

Interestingly, the synthesis of TV involves the nucleophilic incorporation of indole to TM. Notably, a *K. oxytoca* Δ*tnaA* mutant’s inability to produce indole leads to the absence of TV synthesis. However, the cytotoxic activity of the Δ*tnaA* mutant is very similar to the wild-type strain, indicating that TM alone is sufficient to cause damage to epithelial cells. Indeed, it has been demonstrated that TM is more cytotoxic than TV (6). TM and TV are crucial to produce the AAHC. Both enterotoxins are non-ribosomal peptides that impede cell cycle progression. TM causes cellular DNA damage, arresting the cells at the G1/S phase, and TV interacts with tubulin, impeding microtubule disassembly by its stabilization, which arrests the cells at the G2/M phase (9). Both TM and TV cause apoptosis (6) and damage the tight junction protein claudin-1, leading to an impaired intestinal barrier in the colonic epithelium cells (10).

Bacteria respond to changes in environmental conditions by regulation of transcription. Indeed, bacteria must rapidly sense and respond to their environment to optimize fitness, adapt, and express their virulence factors in the case of pathogenic bacteria. The global regulator Lrp (*l*eucine-responsive *r*egulatory *p*rotein) is one of the transcription factors that control such responses (11–13). Previously, we found that *aroX*- and NRPS-operons transcription is activated directly by the global regulator CRP (cAMP receptor protein), where the regulation via CRP is boosted by lactose (14). Nevertheless, the role of other global regulators in the expression of *aroX*- and NRPS-operons remains unknown.

Lrp is one of the top seven global regulators involved in the modulation of various metabolic and physiological functions and is widely distributed among prokaryotes and archaea. In *Escherichia coli*, Lrp regulates the expression of approximately one-third of its genome; the overall regulatory behavior of Lrp extends to 38% of *E. coli* genes, including some involved in amino acid metabolism and transport (15, 16). Lrp is an 18.8 kDa DNA-binding protein that can act as a positive and negative transcriptional regulator by directly or indirectly binding to the regulatory regions of target genes (16–20). l-leucine can augment its activity; indeed, it has been shown that Lrp self-associates mainly into octamers, and such oligomeric structure could be enhanced by the presence of l-leucine (21–24). Additionally, Lrp monitors a general nutritional state by sensing the concentrations of l-leucine in the cell and regulating genes involved in entering the stationary growth phase in *E. coli*. In fact, Lrp production is increased during the stationary phase (25, 26).

In the context of histopathological lesions, it is crucial to understand that *K. oxytoca* colonizes the large intestine, which has significant implications for our health. The large intestine, characterized by its nutrient-poor environment due to the absorption processes of the small intestine, may trigger the production of the Lrp regulatory protein. This protein plays an essential role in managing the transition from nutrient-rich to nutrient-poor conditions, highlighting the complex interplay between our gut microbiota and overall well-being (27, 28).

In this work, we set out to uncover the pivotal role of Lrp in the transcriptional regulation of the TM/TV biosynthetic genes, both in the absence and presence of l-leucine. Furthermore, we explored the influence of Lrp on the cytotoxic effect of *K. oxytoca* on HeLa cells. Our study not only sheds light on the novel function of Lrp as a transcriptional activator of genes involved in the biosynthesis of *K. oxytoca* TM and TV enterotoxins but also sets the stage for further research into bacterial pathogenicity.

## MATERIALS AND METHODS

### Bacterial strains and growth conditions

We used the *K. oxytoca* toxigenic strain MIT 09-7231 (29), its derivative Δ*lrp* mutant and the complemented strain Δ*lrp* pT3-Lrp. All the strains and plasmids used in this work are further listed in Table 1. Tryptone soy broth (TSB) (Difco), and N-minimal medium (N-MM) (5 mM KCl, 7.5 mM (NH_4_)_2_SO_4_, 0.5 mM K_2_SO_4_, 1 mM KH_2_PO_4_, 100 mM Tris-HCl, 10 µM MgCl_2_, 0.5% glycerol, and 0.15% casamino acids) at pH 7.2 (30), were used to grow cultures at 37°C. Culture media were supplemented with 100 µg/mL l-leucine (Sigma) when indicated (31). When necessary, antibiotics were added: ampicillin (200 µg/mL), kanamycin (50 µg/mL), or tetracycline (10 µg/mL). For all the *K. oxytoca* strains studied in this work, we used cultures at an OD_600nm_ of 1.6, a method based on our previous study that demonstrated a significant increase in the gene expression of *aroX*- and NRPS-operons in the stationary growth phase (14). This reliability in our methods is further reinforced by our exploration of Lrp, a transcriptional regulator that plays an essential role in the stationary phase of cell growth by coordinating metabolic shifts between nutritional feast and famine conditions (25, 32, 33).

**TABLE 1:** Bacterial strains and plasmids used in this study^*a*^

Strain or plasmid	Description	Reference
Strains		
*K. oxytoca* WT	Wild-type *K. oxytoca* strain MIT 09–7231	(29)
*K. oxytoca* Δ*lrp*	*K. oxytoca*Δ*lrp*::Km^R^	This study
*K. oxytoca* Δ*npsA*	*K. oxytoca*Δ*npsA*::FRT	(14)
*E. coli* BL21 (DE3)	F^−^*omp*T *hsd*S_B_ (r_B_^−^, m_B_^−^) *gal dcm* (DE3)	Invitrogen
*E. coli* MC4100	Cloning strain F^−^ *araD139*Δ(*argF-lac*) *U169 rspL150 relA1 flbB5301 fruA25 deoC1 ptsF25*	(34)
Plasmids		
pMPM-T3	p15A derivative low-copy-number expression vector, *lac* promoter; Tc^R^	(35)
pT3-Lrp	pMPM-T3 derivative expressing *lrp* from the *lac* promoter, Tc^R^	This study
pMPM-T6	p15A derivative expression vector, pBAD (*ara*) promoter; Tc^R^	(35)
pT6-Lrp	pMPM-T6 derivative expressing N-terminal His_6_-Lrp from pBAD (*ara*) promoter, Tc^R^	This study
pKD119	pINT-ts derivative containing the λ Red recombinase system under an arabinose-inducible promoter, Tc^R^	(36)
pKD4	pANTsy derivative template plasmid containing the kanamycin cassette for λ Red recombination, Ap^R^	(36)

^*a*^ Km^R^, kanamycin resistance; Tc^R^, tetracycline resistance; Ap^R^, ampicillin resistance.

### Lrp protein sequences analysis

Amino acid sequence alignment of Lrp from *K. oxytoca* MIT 09-7231 (GenBank accession number: KMV84644.1 with other selected *Enterobacterales* Lrp homologs was carried out using Clustal Omega (https://www.ebi.ac.uk/Tools/msa/clustalo/) (37).

### Generation of the *lrp* isogenic mutant strain

The *lrp* gene was deleted using the one-step mutagenesis procedure, replacing the target gene with a selectable kanamycin resistance gene marker with the λ-Red recombinase system, as previously described (36). The mutation was characterized by PCR and sequencing to confirm its authenticity.

### Generation of the mutant probes in the Lrp putative motifs

Three probes with substitution point mutations in the two Lrp putative motifs located in the intergenic regulatory region of the divergent *aroX*- and NRPS-operons were obtained using overlapping PCR (38). This process consists of two rounds of PCR with specialized primers (Table 2). In the first PCR, we generated two fragments for each probe: one with the 5′-half and another with the 3′-half of such intergenic region, including the overlapping mutated region. Afterward, we combined the two fragments and amplified them in a second PCR to obtain the overlapping product. Finally, a DNA sequencing process was performed to authenticate the substitution point mutations, ensuring the reliability and accuracy of our results.

**TABLE 2:** Primers used in this study^*a*^

Primer	Sequence (5′−3′)	Target gene
For gene deletion		
lrp-H1P1	ATTTTTCTCCTGAGGCGCCCAATGTTAGATAAAA TTGATCGTAAGTGTAGGCTGGAGCTGCTTCG	*lrp*
lrp-H2P2	AATTCGTAGCGGCTGCTGCCGTTAATCAATAGGC AAGGCTGTGGTCATATGAATATCCTCCTTAG	
For gene deletion characterization		
lrp-MC-F	TAGCCTTTCAATTACAATAAAAGGAGT	*lrp*
lrp-MC-R	ATCGCAAGCAGTTAAATCCTGA	
For site-directed mutagenesis		
aronpLrpBox5′-F	TCTCTCACTCGAAATTTAACAGGT	*aroX-npsA*
aroXLrpBox1-F	TTTTTTTTTGG**GTAGCCTTCCGCGAC**AGGGTGAGGTT	*aroX-npsA*
aroXLrpBox1-R	AACCTCACCCT**GTCGCGGAAGGCTAC**CCAAAAAAAAA	
npsALrpBox2-F	ATTGATTTTT**ATCGTGTAAGACCATGAT**TGTTTTTT	*aroX-npsA*
npsALrpBox2-R	AAAAAACAATC**ATGGTCTTACACGAT**AAAAATCAAT	
aronpLrpBox1/2F	ATTTTT**ATGGTTATTATTTTT**GATTGTTTTTTATTTTTTGCTATATGATTTTTTTTTGG**AAATCAAATTGACTG**AGGGTGAGGTT	*aroX-npsA*
aronpLrpBox1/2F	AACCTCACCCT**CAGTCAATTTGATTT**CCAAAAAAAAATCATATAGCAAAAAATAAAAAACAATC**AAAAATAATAACCAT**AAAAAT	
aronpLrpBox3′-R	TCTCTCCTGGAGAATTAGGAACG	*aroX-npsA*
For expression vector constructions		
lrp-XhoI-F	GGG*CTCGAG*GAACCCAGGATAAGCAGTTTACTG	*lrp*
lrp-EcoRI-R	CCC*GAATTC*GATGCTAAAACGGACAACAGTAC	
lrp-NcoI-F	GGG*CCATGG*TACATCATCATCATCATCAT GATAGCAAGAAGCGCCCTGG	*lrp*
lrp-HindIII-R	CCC*AAGCTT*TCAGCTAAAACGGACAACAGTG	
For qPCR		
aroX-F	TGTTGCCTGCAAGATTGACG	*aroX*
aroX-R	ATGTGTGAACGGCCAAAACG	
npsA-F	AAATACGTGGCTTCCGCATC	*npsA*
npsA-R	TCCTGCGTGACATAACAAGC	
rrsH-F	CAGGGGTTTGGTCAGACACA	*rrsH*
rrsH-R	GTTAGCCGGTGCTTCTTCTG	
For EMSA		
aroX-npsA-F	TCTCTCACTCGAAATTTAACAGGT	*aroX-npsA*
aroX-npsA-R	TCTCTCCTGGAGAATTAGGAACG	
ilvI-F	CGTTGAAGGTCAATAGCCGT	*ilvI*
ilvI-R	ATCTCCATGGTTTGCCTCCC	
fbpA-F	TTCCTGACCAGCGAGCTGCCG	*fbpA*
fbpA-R	CCCCAGTACTCCCAGCTGTGC	
aronpsLrpF1-F	TCTCTCACTCGAAATTTAACAGGT	*aroX-npsA*
aronpsLrpF1-R	TTTTGATTGACATAATGCACTCATTGG	
aronpsLrpF2-F	AAATTTATATTTGATTTATGTTTTT	*aroX-npsA*
aronpsLrpF2-R	AAATTTATATTTGATTTATGTTTTT	
aronpsLrpF3-F	AGTTATGTTTGTGTTGCTTA	*aroX-npsA*
aronpsLrpF3-R	TCTCTCCTGGAGAATTAGGAACG	

^*a*^ The sequence corresponding to the template plasmid pKD4 is underlined. Italic letters indicate the respective restriction enzyme site in the primer. Bold letters show the overlapping section to introduce the substitution site-directed mutage.

### Construction of plasmids

We constructed the plasmids pT3-Lrp and pT6-Lrp to complement the Δ*lrp* mutant strain and obtain the His_6_-Lrp recombinant protein, respectively. PCR-purified *K. oxytoca lrp* gene products were generated with specific primers (Table 2). The pT3-Lrp construction was achieved by cloning the *lrp* gene into the pMPM-T3 vector with XhoI and EcoRI restriction enzymes. The pT6-Lrp was created by cloning the *lrp*, containing a nucleotide sequence that encodes a His_6_-tag at the N-terminal of the Lrp recombinant protein, into the pMPM-T6 vector with NcoI and HindIII restriction enzymes. These constructs were verified by DNA sequencing.

### RNA isolation and reverse transcription-quantitative PCR

Total RNA was isolated from cultures in the stationary phase (OD_600nm_ of 1.6) using the hot phenol method (39). RNA was purified with the TURBO DNA-free kit (Invitrogen), and its concentration and purity were determined using the NanoDrop ONE (Thermo Scientific). RNA integrity was evaluated by electrophoresis in a bleach-denaturing 1.5% agarose gel (40). For the synthesis of cDNA, the reliable RevertAid First Strand cDNA Synthesis Kit (Thermo Scientific) was used with 1 µg of RNA as a template. Quantitative PCR was carried out in a LightCycler 480 instrument (Roche) with specific primers (Table 2), as previously described (14). The *rrsH* gene, which codes for 16S rRNA was used for normalization, and experiments were performed in triplicate from three independent assays. Minus RT controls were included in all experiments. The 2^−ΔΔC*t*^ formula (41, 42) was used to calculate the relative gene expression.

### Purification of Lrp protein

The His_6_-Lrp expression vector pT6-Lrp was electroporated into a competent *E. coli* BL21 (DE3) strain, and the recombinant protein was efficiently purified as previously described (43). The process, designed for maximum efficiency, began with the growth of bacteria containing the recombinant plasmid to the mid-exponential phase. L(+)-arabinose was added at final concentration of 1% and cultured for 6 h at 37°C. After centrifugation, the cellular pellet was resuspended in urea buffer (8 M urea, 100 mM Na_2_HPO_4_, and 10 mM Tris-HCl at pH 8.0) and sonicated. The lysate was pelleted by centrifugation, and the supernatant was filtered through a 0.22 µM nitrocellulose filter unit (Merck Millipore) and purified through a Ni-NTA agarose column (Qiagen). After adding 200 mL of washing buffer (50 mM imidazole, 10 mM Na_2_HPO_4_, 1.8 mM KH_2_PO_4_, 137 mM NaCl, and 2.7 mM KCl at pH 7.4), the protein was eluted by adding 10 mL of eluting buffer (500 mM imidazole, 10 mM Na_2_HPO_4_, 1.8 mM KH_2_PO_4_, 137 mM NaCl, and 2.7 mM KCl at pH 7.4).

Analysis of fractions was performed using SDS-PAGE and Coomassie blue staining. Protein concentration was calculated using the Bradford method (Bio-Rad). The recombinant His_6_-Lrp purified protein was stored at −70°C in a sterile storage buffer solution (50% Glycerol, 10 mM Na_2_HPO_4_, 1.8 mM KH_2_PO_4_, 137 mM NaCl, and 2.7 mM KCl at pH 7.4).

### Recognition of promoters and Lrp binding sites on the *aroX-npsA* intergenic region

The prediction of promoters on the *aroX* and *npsA* regulatory regions was determined by analyzing 430 nucleotides upstream to the initial codon of both genes. We used the genomic sequence from *K. oxytoca* MIT 09-7231 (GenBank accession number: GCA_001078175.1) and the web-based program Neural Network Promoter Prediction (https://www.fruitfly.org/seq_tools/promoter.html). The Lrp putative motifs were identified using the web-based software PRODORIC (https://www.prodoric.de/) and software Softberry BPROM (http://www.softberry.com/berry.phtml?topic=bprom&group=programs&subgroup=gfindb).

### Electrophoretic mobility shift assay

Electrophoretic mobility shift assay (EMSA) experiments were carried out as previously described (14). Four 448 bp DNA probes corresponding to the regulatory intergenic region of the divergent *aroX* and *npsA* genes were used at a final concentration of 0.2 µM each. The first probe corresponded to the wild-type version of the intergenic region. In contrast, the other three corresponded to substitution mutant versions in the Lrp putative motifs for the transcription of *aroX* and *npsA*. In addition, we used three 150 bp DNA probes corresponding to three different sub-fragments of the intergenic region: (i) the region adjacent to the *aroX* gene, (ii) the central region, and (iii) the region adjacent to the *npsA* gene. These were also used at a final concentration of 0.2 µM each. The probes were mixed with increasing concentrations of His_6_-Lrp (0.0–0.8 µM) with 7.0 mM l-leucine (44) in the gel-shift binding buffer H/S 1× (40 mM HEPES, 8.0 mM MgCl_2_, 50 mM KCl, 1.0 mM DTT, 0.05% NPSA, and 0.1 mg/mL BSA).

As positive and negative controls, DNA probes from the regulatory region of the *K. oxytoca ilvI* gene and the coding region of the *Mycobacterium tuberculosis fbpA* gene were used. Reactions were incubated at room temperature for 20 min and then separated by electrophoresis in 6% non-denaturing polyacrylamide gels using 0.5× Tris-borate-EDTA buffer. The DNA bands were stained with ethidium bromide and visualized under UV light.

### LDH cytotoxicity assays

A lactate dehydrogenase (LDH) cytotoxicity assay kit (Invitrogen) was used according to the manufacturer’s instructions to measure LDH released from HeLa cells after damage to plasma membrane integrity. About 10 µL of negative control (PBS), culture medium control (TSB), positive control (lysis buffer), and filtered bacterial supernatants (wild-type, Δ*lrp*, Δ*lrp* pT3-Lrp, and Δ*npsA*) from cultures that had reached an OD_600nm_ of 1.6. were added to 1 × 10^4^ HeLa cells previously cultivated in 100 µL DMEM high glucose (4.5 g/L) (Invitrogen) with 10% FBS (Gibco) into a 96-well flat-bottom plate culture. Then, it was incubated at 37°C under a 5% CO_2_ atmosphere for 48 h. Afterward, 50 µL each sample medium was transferred to a new 96-well plate, and kit solutions were added into each well. The absorbance was measured at 490 and 680 nm with a spectrophotometer (Multiskan Ascent, Thermo Scientific). The experiment was performed in three independent biological replicates by triplicates, and results were expressed as LDH cytotoxicity by deducting the 680 nm absorbance background value from the 490 nm absorbance value.

### Statistical analysis

An unpaired two-tailed Student’s *t* test was performed with the GraphPad Prism 9.0 software (GraphPad Inc., San Diego, CA, USA) for statistical differences. All data were obtained from three independent experiments performed in triplicate, and values of *P* < 0.005 were considered significant.

## RESULTS

### Lrp amino acid sequence analysis

**Fig 1:**
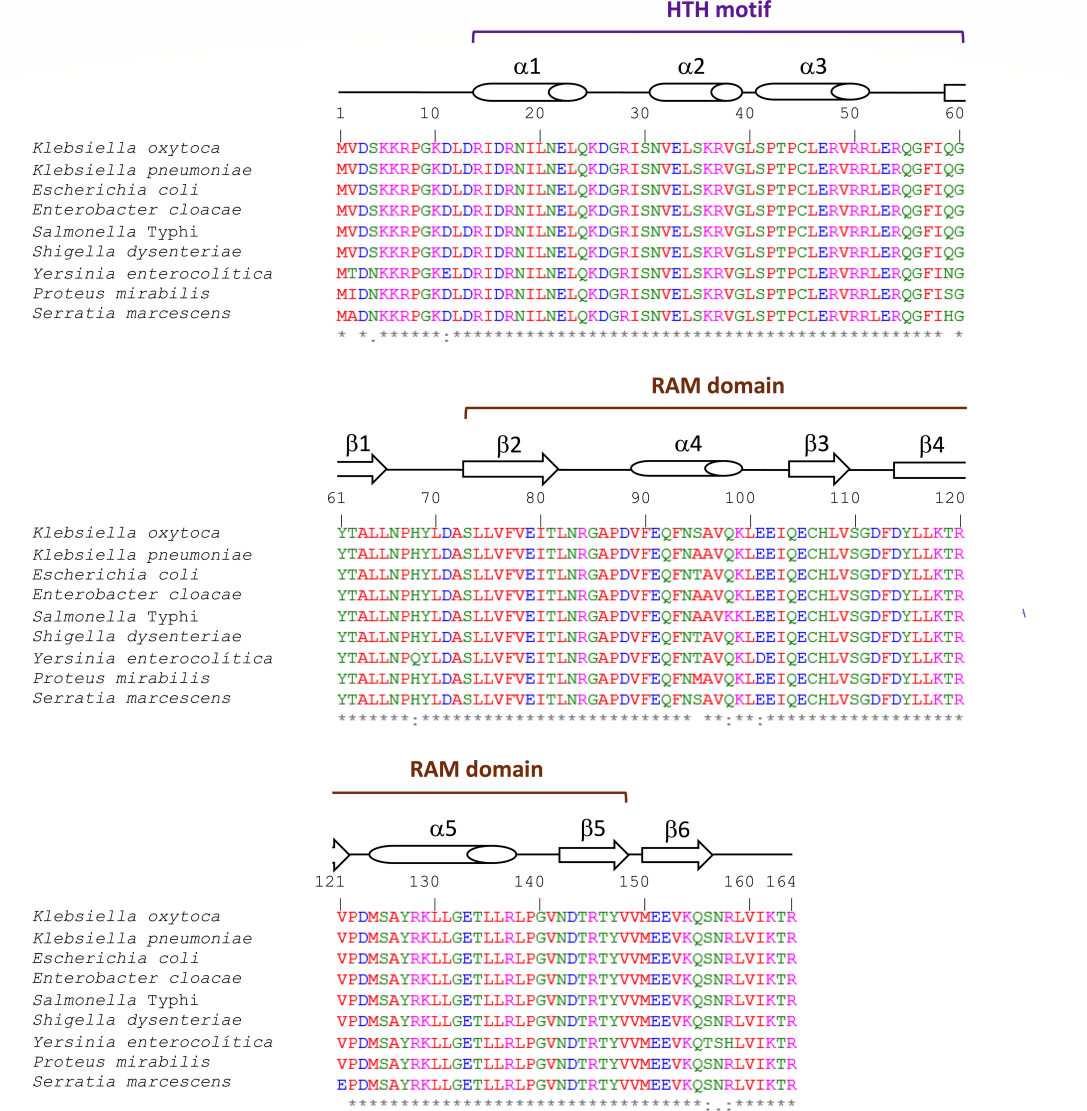
Amino acid sequence alignment of Lrp from *K. oxytoca* and other *Enterobacterales*. The amino acid sequence of Lrp (KMV84644.1) from *K. oxytoca* MIT 09-7231, and Lrp homologs proteins: (BAH62617.1) from *K. pneumoniae* NTUH-K2044, (NP_415409.1) from *E. coli* K-12 MG1655, (ADF62302.1) from *Enterobacter cloacae* ATCC 13047, (AA069588.1) from *Salmonella enterica* serovar Typhi Ty2, (ABB62447.1) from *Shigella dysenteriae* Sd197, (CAL11603.1) from *Yersinia enterocolitica* 8081, (CAR41630.1) from *Proteus mirabilis* HI4320, and (CDG11553.1) from *Serratia marcescens* Db11 were aligned by using Clustal Omega (https://www.ebi.ac.uk/Tools/msa/clustalo/). The predicted regions to encode the HTH motif (DNA-binding), and RAM domain (Regulation of amino acid metabolism) are indicated. Secondary structural elements are shown by barrels (α-helix) and arrows (β-sheet). Hydrophobic, polar, positively charged, and negatively charged amino acids are represented in red, green, magenta, and blue, respectively. The asterisk (*), colon (:), and dot (.) indicate identical, conserved, and semi-conserved amino acids among all aligned sequences.

We initiated this study by comparing the amino acid sequences of a group of *Enterobacterales* Lrp proteins with the *K. oxytoca* toxigenic strain MIT 09-7231 Lrp protein. *K. oxytoca* Lrp amino acid sequence shared an average of 98.24% identity with the other Lrp sequences of *K. pneumoniae*, *E. coli*, *Enterobacter cloacae*, *Salmonella* Typhi, *Shigella dysenteriae*, *Yersinia enterocolitica*, *Proteus mirabilis*, and *Serratia marscesens* (Fig. 1). The putative helix-turn-helix (HTH) and the βαββαβ-fold motifs were identified at the N- and C-terminal of *K. oxytoca* Lrp protein, respectively, through comparison with *E. coli* Lrp (45) by using the web-based PROSITE bioinformatic tool (http://prosite.expasy.org). When comparing the amino acid sequences of Lrp from *K. oxytoca* and *E. coli*, 99.39% identity and 100% similarity were obtained since there is only one change in residue 95 of the protein, being serine for *K. oxytoca* Lrp and threonine for *E. coli* Lrp, both amino acids with uncharged polar side chains containing aliphatic hydroxyl groups.

### Lrp is required for optimal growth of *K. oxytoca* under nutrient-limiting conditions

**Fig 2:**
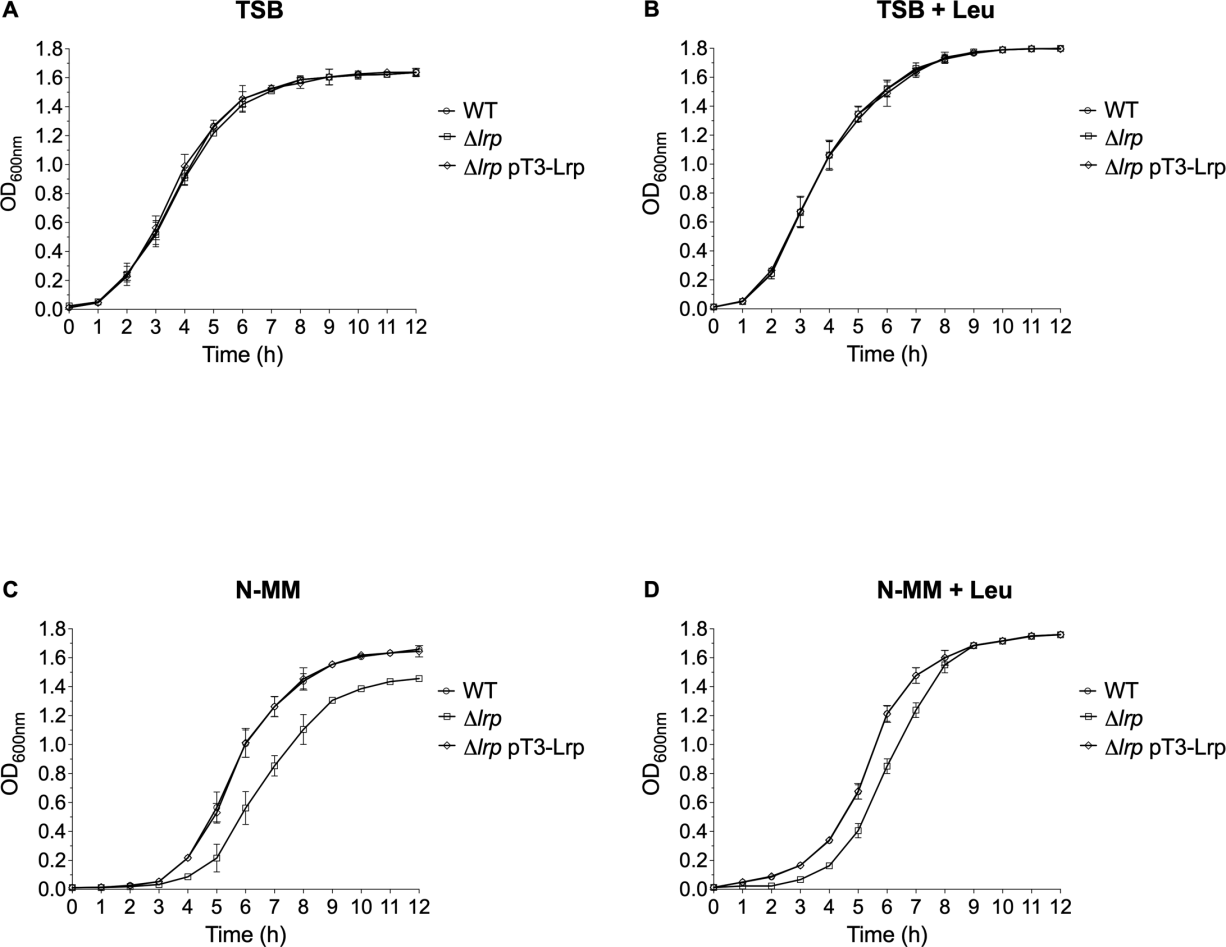
Growth curves of *K. oxytoca* wild-type (WT), Δ*lrp* mutant, and Δ*lrp* pT3-Lrp complemented strains. Cultures were grown for 12 h in TSB (nutrient-rich) (**A, B**) and N-MM (nutrient-limiting) (**C, D**) medium, in the absence and presence of l-leucine (Leu). The OD_600nm_ values were recorded every hour. Data represent the mean of three independent experiments with standard deviations.

To find out whether *K. oxytoca* toxigenic strain MIT 09-7231 Lrp participates in the regulatory process related to growth, we compared growth rates in the wild-type, its derivative Δ*lrp* mutant, and complemented strains in two different culture media: TSB (nutrient-rich) and N-MM (nutrient-limiting) in the absence and presence of 100 µg/mL l-leucine (31). No growth differences existed between the wild-type, Δ*lrp*, and complemented strains in TSB without or with leucine (Fig. 2A and B). In contrast, in N-MM without leucine, the Δ*lrp* strain was attenuated in exponential and stationary phases (Fig. 2C). Adding leucine to N-MM restored the growth defect in the stationary phase (Fig. 2D). Furthermore, our comparison of the growth in TSB without or with leucine revealed that the growth of all the strains was enhanced by adding leucine to the growth medium from 7 to 12 h (Fig. 2A and B). Intriguingly, in N-MM, the growth of all the strains was improved by adding leucine from 1 to 12 h (Fig. 2C and D). These observations indicate that Lrp is involved in *K. oxytoca* toxigenic strain MIT 09-7231 growth regulatory processes as in other bacteria (26, 46–48). Furthermore, adding leucine improved growth, suggesting that this amino acid may play an essential role in maintaining central metabolism in *K. oxytoca*, as reported in other microorganisms (49–51).

### Lrp is an activator of *aroX* and *npsA* gene transcription

**Fig 3:**
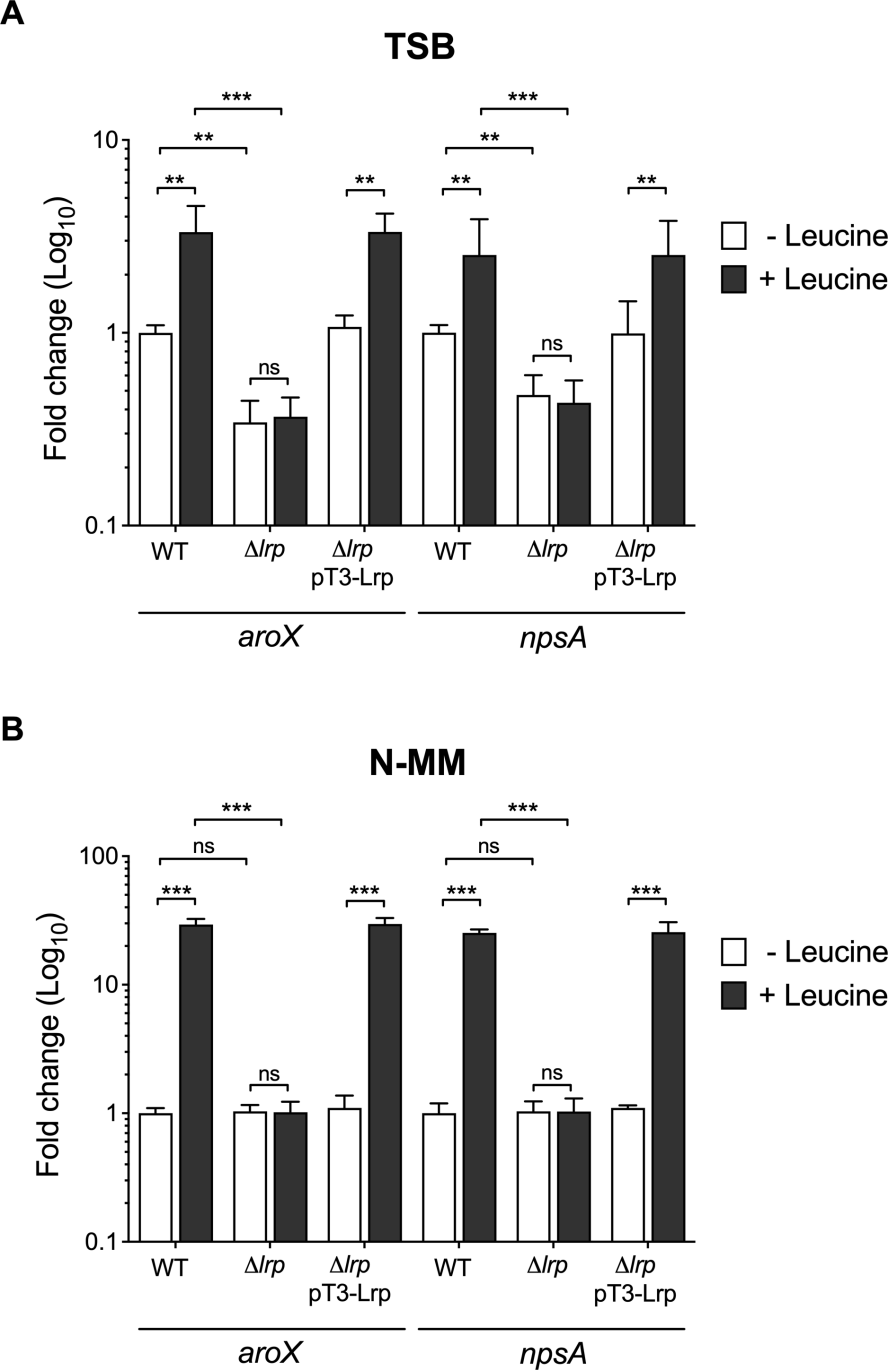
Transcriptional regulation of Lrp on *aroX* and *npsA* genes. Determination of gene expression by RT-qPCR of *aroX* and *npsA* of *K. oxytoca* wild-type (WT), Δ*lrp* mutant, and Δ*lrp* pT3-Lrp complemented strains grown in TSB (nutrient-rich) and N-MM (nutrient-limiting) medium at stationary phase (OD_600nm_ = 1.6) at 37°C, in the absence and presence of l-leucine. Data represent the mean of three independent experiments performed in triplicate with standard deviations. Statistically significant: **P < 0.01; ***P < 0.001; ns: not significant. All *P* values were determined using unpaired two-tailed Student’s *t* test.

To investigate the regulatory activity of Lrp on *aroX* and *npsA* transcription, expression of such TM/TV genes was determined by RT-qPCR in *K. oxytoca* toxigenic strain MIT 09-7231 and its derivatives mutant Δ*lrp* and complemented strains under the stationary growth phase (OD_600nm_=1.6) in TSB (nutrient-rich) and N-MM (nutrient-limiting) culture media. In TSB without leucine, the transcriptional levels of both *aroX* and *npsA* genes were downregulated three- and twofold, respectively, in the Δ*lrp* strain compared to the wild-type (Fig. 3A). However, adding leucine to TSB significantly enhanced the transcription of *aroX* and *npsA* 3.3- and 2.5-fold, respectively, in the wild-type strain, shedding light on the crucial role of leucine in gene transcription. Interestingly, the transcriptional levels of both TM/TV genes were not significantly different in the Δ*lrp* strain either in the absence or the presence of leucine (Fig. 3A).

In N-MM without leucine, our research revealed that the transcription of *aroX* and *npsA* was not affected in the Δ*lrp* strain compared to the wild-type strain. However, when leucine was added to N-MM, the transcription of both TM/TV genes was upregulated more than 25-fold in the wild-type strain (Fig. 3B). As in TSB, the transcription of *aroX* and *npsA* was not altered in the Δ*lrp* strain in presence of leucine (Fig. 3B). In both culture media, TSB and N-MM, the transcription of *aroX* and *npsA* was boosted in the wild-type strain after the addition of leucine, while the transcription of both TM/TV genes did not change in the Δ*lrp* strain in the presence of leucine. In N-MM, the positive regulatory activity of Lrp was only observed by the addition of leucine (Fig. 3A and B).

These results, along with the consistent restoration of the transcription of the complemented strain harboring the pT3-Lrp low-copy number plasmid to wild-type levels, underscore the robustness and reliability of our findings. We have demonstrated that Lrp positively regulates the transcription of *aroX* and *npsA* genes, and that leucine induces Lrp-mediated *aroX* and *npsA* transcription.

### Identification of putative promoters and Lrp binding motifs on the *aroX-npsA* intergenic region

**Fig 4:**
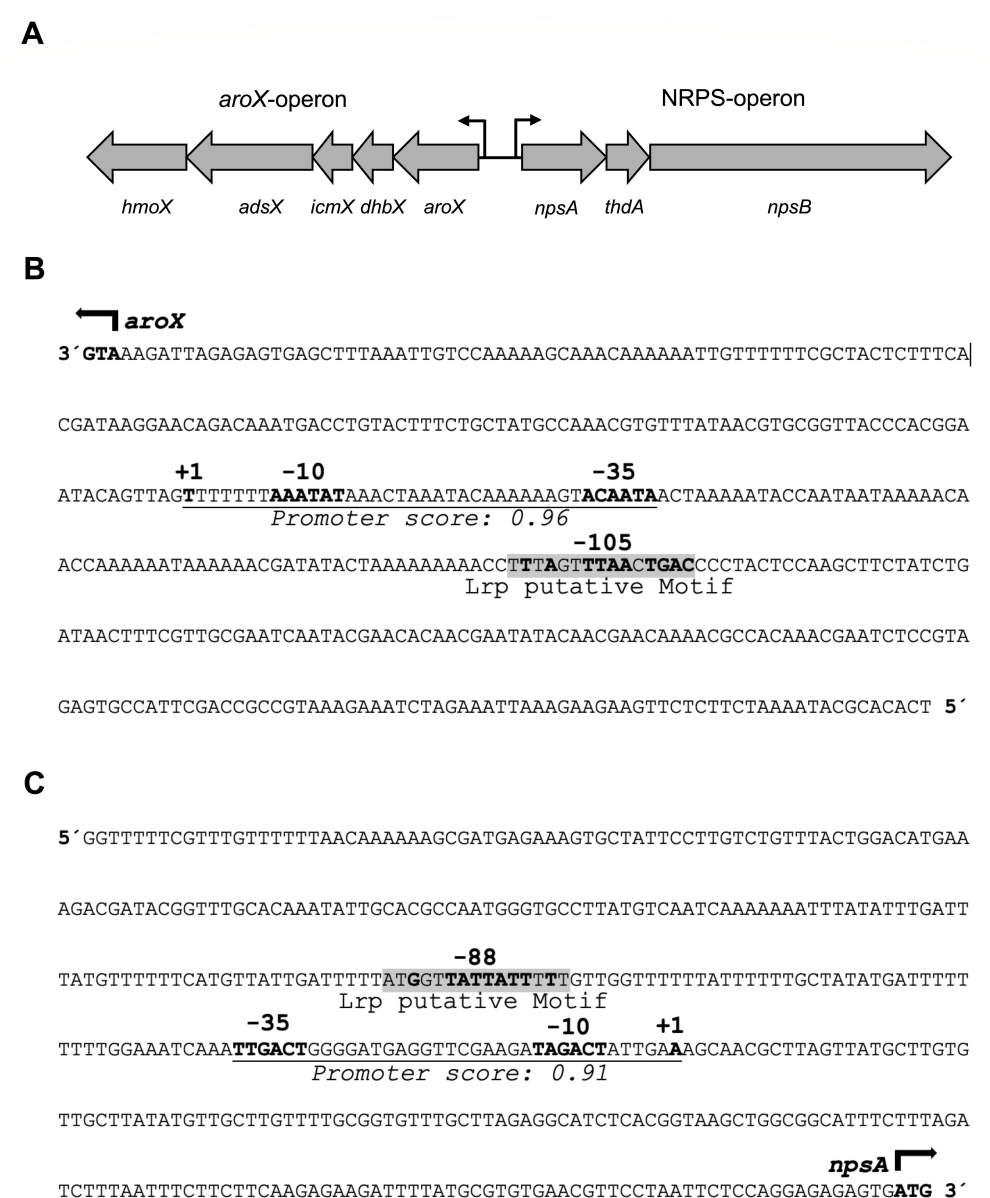
*In silico* analysis of the intergenic region of *aroX* and *npsA* genes. (**A**) Genetic organization of the *aroX* and NRPS operons. Regulatory regions of *aroX* (**B**) and *npsA* (**C**) indicating the initiaton codon (ATG, bold), the predicted promoter region (−35 and −10, bold), the transcription start site (+1, bold), and the Lrp-binding motif (highlighted in gray; nucleotides matching the consensus sequence from *E. coli* are bold).

The cytotoxin biosynthetic gene cluster is arranged in two divergent operons, *aroX* and NRPS (Fig. 4A). The *aroX*-operon is constituted by the *aroX*, *dhbX*, *icmX*, *adsX*, and *hmoX* genes, whereas the NRPS operon is constituted by the *npsA*, *thdA*, and *npsB* genes (6, 7, 14). *In silico* analyses were performed to identify regulatory motifs in the intergenic region of *aroX* and *npsA* genes. We identified putative promoters for the *aroX* and *npsA* genes located 151 and 163 bp upstream from their coding regions, respectively (Fig. 4B and C). Moreover, two putative Lrp binding motifs were found for both TM/TV operons. The Lrp binding motif for the *aroX* gene was located 105 bp upstream of the putative transcriptions start site (TSS, +1) (Fig. 4B), and for the *npsA* gene, it was located 88 bp upstream of the TSS (Fig. 4C).

These findings provide crucial insights into the regulatory regions of the *aroX* and *npsA* genes, that could have significant implications in transcriptional expression research, thereby enhancing our understanding of gene regulation.

### Lrp binds to the *aroX-npsA* intergenic regulatory region

**Fig 5:**
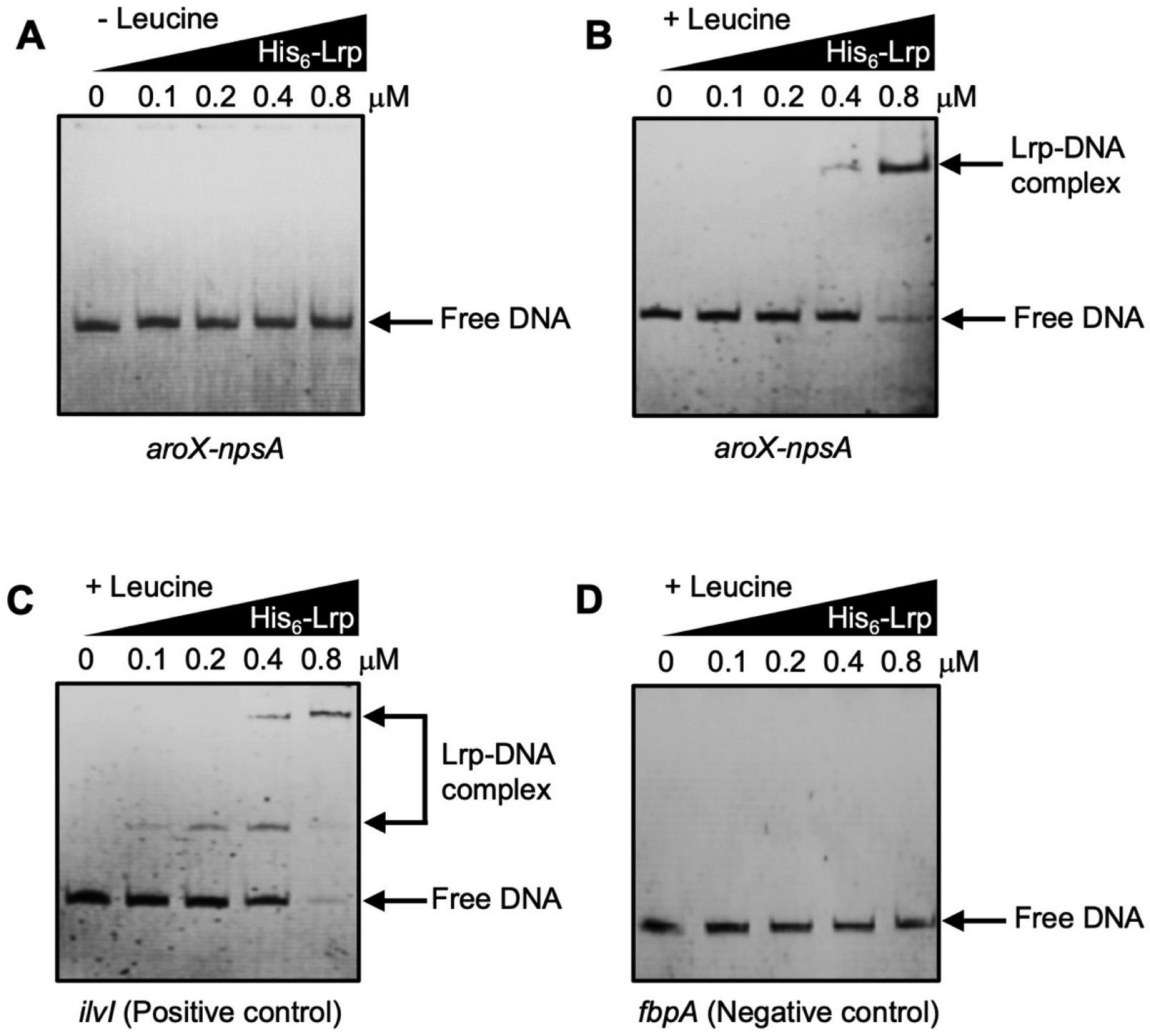
Lrp binds to the intergenic region of *aroX* and *npsA* genes in the presence of leucine. Electrophoretic mobility shift assays (EMSAs) were carried out to find out the binding of the His_6_-Lrp purified recombinant protein to the DNA probe from the intergenic regulatory region of *aroX* and *npsA* in the absence (**A**) or presence (**B**) of leucine. DNA probes from *K. oxytoca ilvI* regulatory region (**C**) and *M. tuberculosis fbpA* coding region (**D**) were employed as positive and negative controls, respectively. 0.2 µM of DNA fragments was individually mixed and incubated with increasing concentrations of purified His_6_-Lrp. l-leucine was added at a final concentration of 7 mM. Arrows show free DNA or Lrp-DNA complex stained with ethidium bromide.

This research was built on a foundation of meticulously designed experiments that have revealed a crucial aspect of the Lrp binding process. We conducted EMSA with a recombinant His_6_-Lrp protein and several DNA fragments. Notably, Lrp bound to the fragment corresponding to the *aroX-npsA* intergenic region only in the presence of l-leucine (7 mM) using 0.4 and 0.8 µM of the recombinant protein (Fig. 5A and B), corroborating the role of leucine on the Lrp DNA-binding activity. To validate our results, DNA fragments of *ilvI* regulatory region (52, 53) and *fbpA* coding region (54) were employed as positive and negative controls, respectively (Fig. 5C and D).

**Fig 6:**
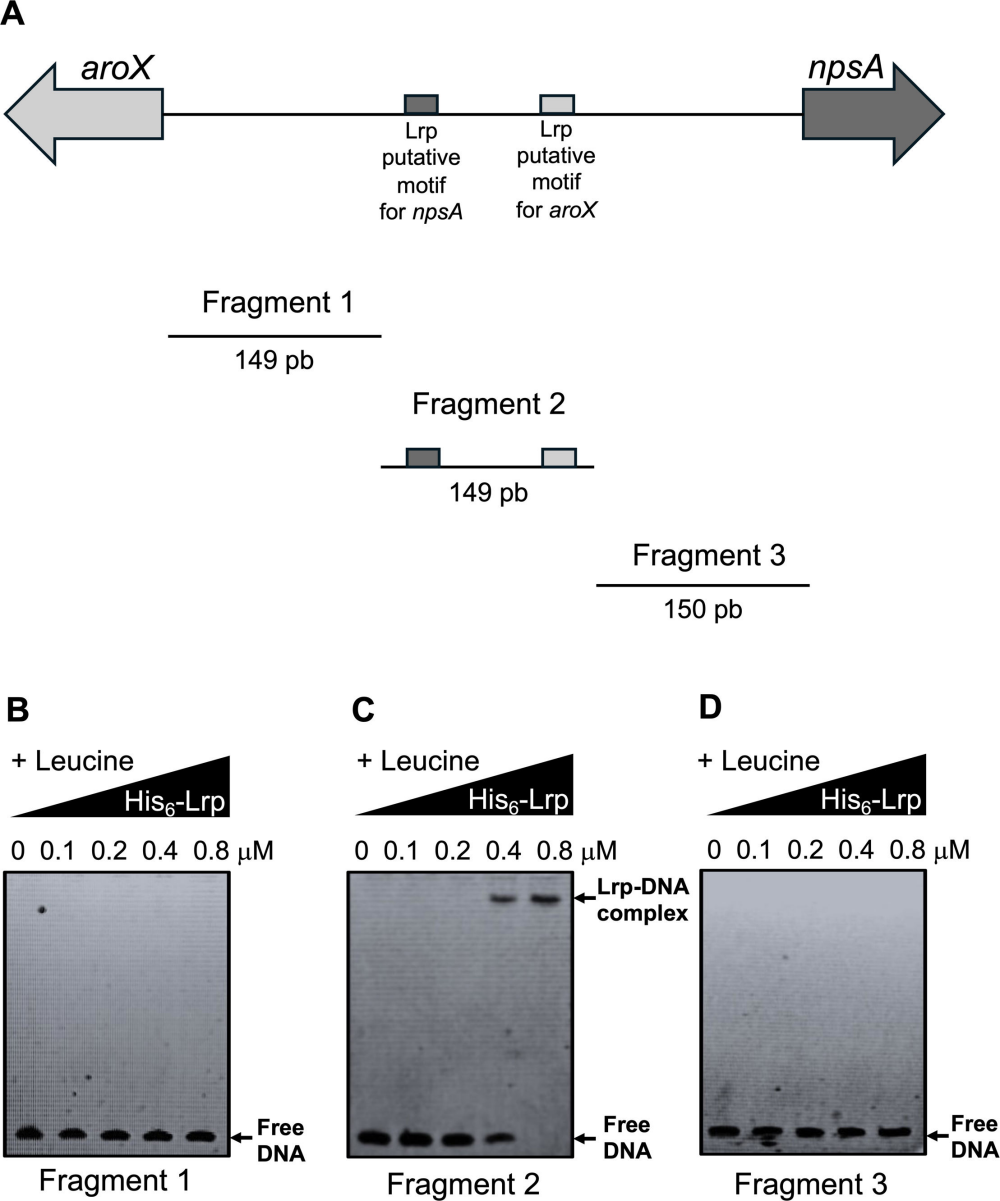
Analysis of sequences to determine the bind of Lrp to the intergenic region of *aroX* and *npsA* genes. Electrophoretic mobility shift assays (EMSAs) were performed to determine the binding of the His_6_-Lrp purified recombinant protein to three different fragments from the intergenic regulatory region of *aroX* and *npsA*. Schematic representation of the *aroX-npsA* intergenic regulatory region indicating the position of the two Lrp putative motifs for *npsA* and *aroX*, respectively, and the three fragments analyzed to determine the Lrp binding to DNA (**A**). DNA probes from fragment 1 (**B**), fragment 2 (**C**) and fragment 3 (**D**). 0.2 µM of DNA fragments were individually mixed and incubated with increasing concentrations of purified His_6_-Lrp. l-leucine was added at a final concentration of 7 mM. Arrows show free DNA or Lrp-DNA complex stained with ethidium bromide.

Our investigation into the role of the Lrp putative motifs in the binding process has yielded significant and enlightening findings. The Lrp protein, a key regulator in gene expression, is known to bind to specific DNA sequences, termed Lrp-binding sites, and influence the transcription of nearby genes. We aimed to establish whether these motifs are necessary for Lrp binding to the *aroX-npsA* intergenic regulatory region. Our initial analysis of the binding zones of Lrp in the presence of l-leucine with three different fragments of the *aroX-npsA* intergenic regulatory region revealed a significant finding (Fig. 6A and B). The fact that Lrp bound to the fragment containing the two Lrp putative motifs underscores their crucial role in the process. In contrast, Lrp did not bind to the upstream and downstream fragments concerning the fragment containing both Lrp putative motifs (Fig. 6B). These results demonstrate that Lrp specifically recognizes the sequences at −105 and − 88 bp of the putative transcription start sites of *aroX* and *npsA* genes, respectively.

**Fig 7:**
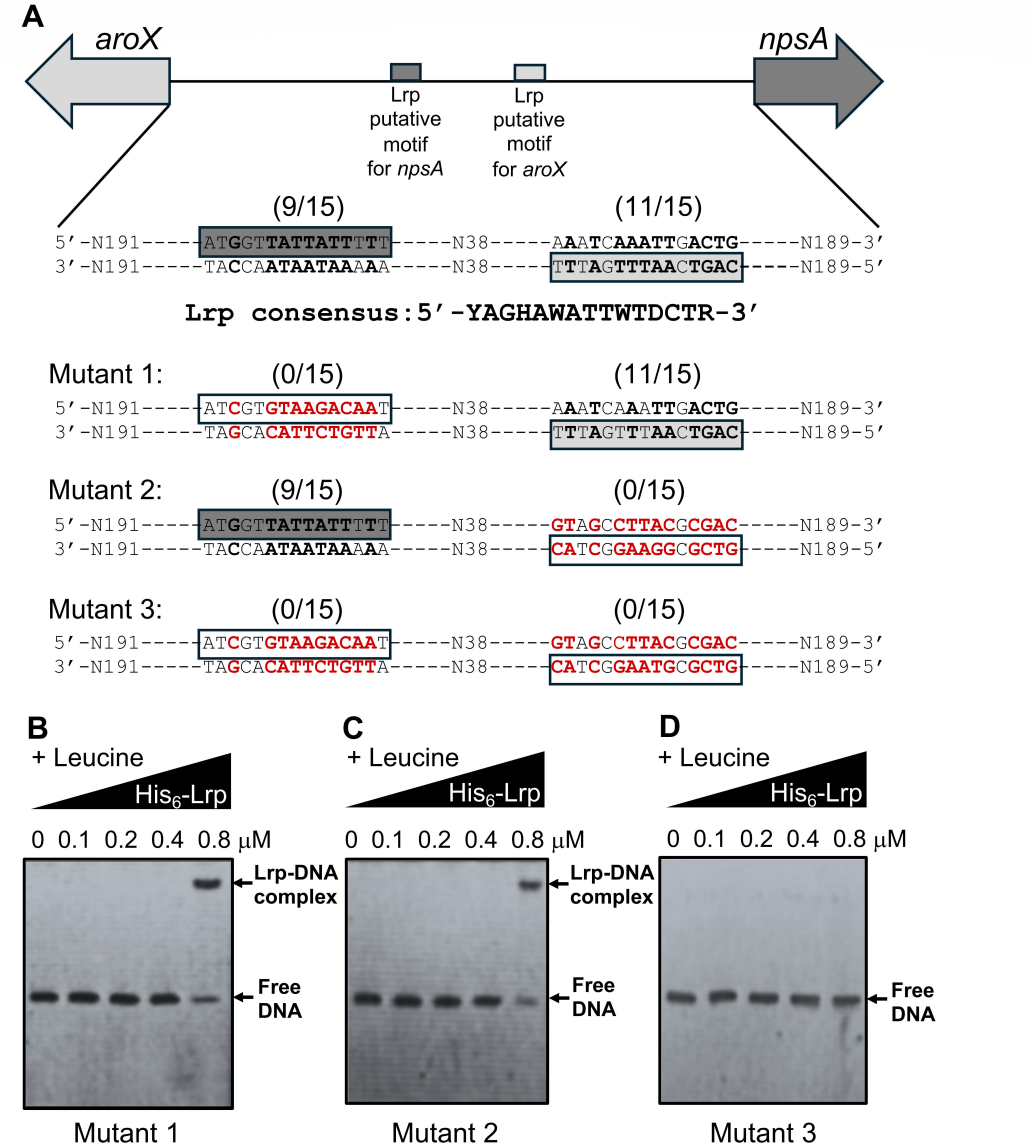
Lrp binds to two specific sequences on the intergenic region of *aroX* and *npsA* genes. Electrophoretic mobility shift assays (EMSAs) were realized to identify the binding of the His_6_-Lrp purified recombinant protein to three fragments from the intergenic regulatory region of *aroX* and *npsA* containing substitution mutations on the Lrp putative motifs. Schematic representation of the *aroX-npsA* intergenic regulatory region indicating the position of the two Lrp putative motifs for *npsA* and *aroX*, respectively, and the three fragments with the different substitution mutations analyzed (**A**). DNA probes from Mutant 1 (**B**), Mutant 2 (**C**) and Mutant 3 (**D**). 0.2 µM of DNA fragments were individually mixed and incubated with increasing concentrations of purified His_6_-Lrp. l-leucine was added at a final concentration of 7 mM. Arrows show free DNA or Lrp-DNA complex stained with ethidium bromide.

Subsequently, we performed substitution site-directed mutagenesis of both motifs to prove whether the two Lrp putative motifs were necessary for the Lrp binding to the *aroX-npsA* intergenic region (Fig. 7). The His_6_-Lrp recombinant protein bound to the probe containing the mutation in the Lrp putative motif for *npsA* (Mutant 1; Fig. 7B) and to the probe containing the mutation in the Lrp putative motif for *aroX* (Mutant 2; Fig. 7C). However, these bindings were only observed when 0.8 µM His_6_-Lrp was used, in contrast to the binding with the wild-type probe, where the shift was observed when 0.4 and 0.8 µM His_6_-Lrp were used (Fig. 5 and 6). These observations indicate that the presence of both putative motifs increases the affinity of Lrp binding to the DNA. In stark contrast, when both putative motifs were mutated, Lrp did not bind to the probe (Mutant 3; Fig. 7D), supporting the direct role of the Lrp protein on the *cis*-sequences found on the *aroX-npsA* intergenic region.

### Lrp promotes the cytotoxic effect of *K. oxytoca* on epithelial cells

**Fig 8:**
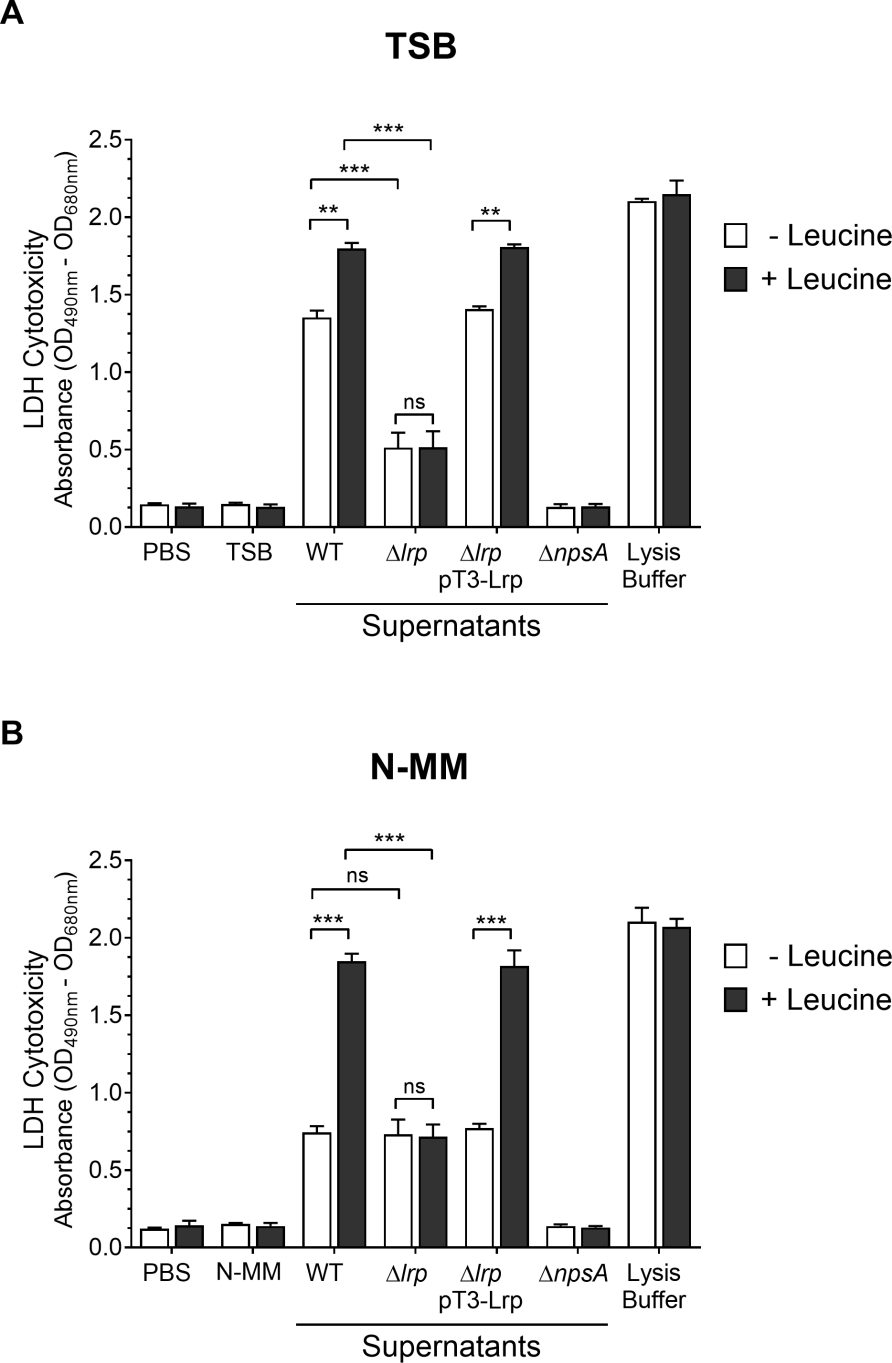
LDH cytotoxicity of *K. oxytoca* WT, Δ*lrp* mutant, and Δ*lrp* pT3-Lrp strains. HeLa cells were inoculated with TSB and N-MM culture media and with *K. oxytoca* supernatants (WT, Δ*lrp*, Δ*lrp* pT3-Lrp, and Δ*npsA*) from cultures with an OD_600nm_ = 1.6, in the absence and presence of leucine, for 48 h. After treatment, the measurement of extracellular LDH was quantified. Minimal and maximal measurable LDH release was determined by incubating HeLa cells with PBS (negative control) and lysis buffer (positive control). Statistically significant: **P < 0.01; ***P < 0.001; ns: not significant. All *P* values were determined using unpaired two-tailed Student’s *t* test.

This work, which involved the use of supernatants from *K. oxytoca* toxigenic strain MIT 09-7231 (wild-type), Δ*lrp* isogenic mutant, and complemented Δ*lrp* strain to conduct LDH release activity assay on HeLa cells, has uncovered novel findings. The wild-type strain supernatants, when used, caused the death of HeLa cells, with the cytotoxicity being higher when bacteria were cultivated in media with leucine (Fig. 8). In contrast, when supernatants of the Δ*lrp* strain were used, the cytotoxicity was remarkably diminished when bacteria were cultivated in TSB, either in the absence or the presence of leucine (Fig. 8A). Interestingly, when bacteria were cultivated in N-MM, the cytotoxicity was significantly reduced only in the presence of leucine as compared to the wild-type strain (Fig. 8B).

To ensure that the cytotoxic activity depends on TM and TV production, we used the Δ*npsA* mutant strain, which does not produce either TM or TV (6, 7). As expected, the Δ*npsA* mutant strain supernatants did not cause the death of HeLa cells, demonstrating, one more time, that the *K. oxytoca* cytotoxic effect on epithelial cells is dependent on TM and TV production. A significant finding of these results is the role of Lrp in activating the expression of TM and TV biosynthetic genes and, consequently, the production of both enterotoxins in *K. oxytoca*.

## DISCUSSION

The global transcriptional regulator Lrp is a feast/famine regulatory protein member. It is present in Gram-negative, Gram-positive, and archaea, and its amino acid sequence is highly conserved in enteric microorganisms (26, 45, 52, 55). In this study, we found that *K. oxytoca* Lrp protein is homologous to other Lrp proteins from several *Enterobacterale*s such as *K. pneumoniae*, *E. coli*, *Enterobacte*r, *Salmonella*, *Shigella*, *Yersinia*, *Proteus*, and *Serratia*. Regarding bacterial fitness, the lack of Lrp did not affect the growth of *K. oxytoca* in a rich medium (TSB). Nevertheless, in minimal medium (N-MM), the growth of the Δ*lrp* mutant strain was delayed in both exponential and stationary phases. Interestingly, the addition of leucine to the culture medium restored the growth of the Δ*lrp* mutant strain in the stationary phase, which agrees with previous reports in *E. coli*, where the growth of a Δ*lrp* mutant strain in the minimal medium was also affected concerning wild-type strain and such growth defect was restored after addition of leucine (46, 47). Besides, as expected, we found that the addition of leucine to TSB or N-MM growth media enhanced the growth rate in all the strains, confirming that leucine is required as a signal molecule for the Lrp protein, which is involved in the regulation of several cellular processes including metabolism (56). Our findings suggest that Lrp in *K. oxytoca*, as in other microorganisms, integrates nutritional signals by regulating the expression of genes required for growth under amino acid starvation conditions (16, 26, 57, 58).

Our exploration of transcriptional regulation has confirmed the essential Lrp’s role. Lrp, traditionally known as an activator or a repressor, orchestrates a comprehensive and intricate response to leucine (55). Our focus has been on the transcription of the *aroX* and *npsA* genes, the initial genes of the *aroX*- and NRPS-operons, which encode the pivotal enzymes for the biosynthesis of TV (7, 50). We conducted the experiments during the stationary growth phase, a strategic choice based on our previous demonstration that the gene expression of *aroX* and *npsA* is notably higher in this phase (14). This crucial discovery formed the basis for our decision to concentrate on a single time point at OD_600nm_ of 1.6, when the stationary phase is achieved, and the expression of these genes is at its zenith. This method ensures that we capture the gene expression at its most significant stage, thereby offering a more precise portrayal of the biological process.

Our results, which demonstrate the activation of the transcription of *aroX* and *npsA* genes by Lrp in the stationary phase, mark a significant departure from our previous understanding of this regulatory process. This finding, which is contingent on the presence of leucine, is in strong agreement with the previously reported regulatory activities of Lrp on other genes such as *oppA*, *sdaA*, *tdh*, *foo*, and *livK* in *E. coli* (19, 23, 54), and *invF* in *S. enterica* serovar Typhimurium (56).

Our investigation validated the pivotal function of Lrp as a transcriptional regulator that plays a crucial role in the stationary phase of cell growth. In *E. coli*, Lrp influences nearly three-quarters of the genes induced when cells enter the stationary phase, including genes that respond to nutrient limitation. Indeed, in a rich medium, Lrp levels decrease during the lag phase, remain constant during the exponential phase, and then increase upon entry into the stationary phase. Intriguingly, in a minimal medium, Lrp is fourfold higher than in a rich medium, suggesting that Lrp and leucine might function to coordinate metabolic shifts between nutritional feast and famine conditions (25, 33). These findings underscore the importance of our research in understanding the complex regulatory mechanisms in molecular biology and microbiology.

Our research, which explores the role of leucine in gene regulation via Lrp, has significant implications. It has been reported that for some genes, leucine binding to Lrp may increase its affinity to promoters on DNA and regulate their expression (58). Leucine binding to Lrp induces conformational changes, favoring the dissociation of a hexadecameric to octameric form by enhancing dimer-dimer interactions and promoting its binding to DNA. These conformational changes are crucial as they alter the structure of Lrp, making it more conducive to binding to DNA and regulating gene expression (31). In our results, the addition of l-leucine to the Lrp recombinant protein was crucial for its DNA-binding activity to the a*roX-npsA* intergenic region, suggesting a concerted mechanism of Lrp regulatory protein as was observed with the *fimAICDFGH* promoter (20, 24). Moreover, the position of Lrp-binding sites located at −105 and − 88 bp of the putative transcription start sites of *aroX* and *npsA* genes, respectively, suggests that Lrp would act as a classic transcriptional activator recruiting to RNA polymerase.

Our data provide a compelling explanation for the upregulation of gene expression of *aroX*- and NRPS-operons and TM/TV biosynthesis during the stationary growth phase, even when nutrients are limited. This effect could be strongly related to Lrp activity, a key player in activating biosynthetic operons. The physiological role of Lrp is to activate these operons and repress degradative ones, which are overexpressed in the stationary phase. Our results confirm that Lrp and l-leucine are crucial in coordinating metabolic shifts between nutritional feast and famine conditions. Lrp mediates transitions between exponential and stationary growth phases due to its reciprocal regulation of amino acid metabolism: biosynthetic genes are activated in the stationary phase. Indeed, the operon *leuABCD*, which is involved in leucine biosynthesis, is activated by Lrp (32). Besides, leucine levels would increase in bacteria during the stationary phase of growth due to the proteolytic degradation of various proteins (59). This process is necessary for bacteria homeostasis and metabolic function during the stationary phase.

These results reveal a novel aspect of the transcriptional activation of *aroX*- and NRPS-operons, strongly suggesting that this activation may be directly mediated by the binding of Lrp to the *aroX-npsA* intergenic region. This finding, which is critically dependent on leucine, underscores the importance of this amino acid in gene regulation. We also demonstrate that Lrp relies on the two identified sites for binding on the *aroX-npsA* intergenic regulatory region. This discovery not only opens exciting possibilities for future research but also holds the potential to significantly advance our understanding of gene regulation mechanisms and offer new avenues for exploration in the field.

The main virulence determinants of toxigenic *K. oxytoca* are the TM and the TV enterotoxins, which cause cytotoxicity in epithelial cells (7, 8, 14, 29, 60–62). Our findings reveal that the absence of Lrp regulatory protein significantly affected the transcription and, subsequently, the secretion of TM and TV, leading to the lack of cytotoxicity of *K. oxytoca* toxigenic strain MIT 09-7231. In terms of genes encoding toxins, Lrp has been described as a positive and negative regulator of *xhlA* and *tcdAB* genes, which encode XhlA hemolysin in *Xenorhabdus nematophila* and toxins A/B in *Clostridioides difficile*, respectively (26, 63).

As a novel research endeavor, our working group will conduct studies with an AAHC mouse model. This model is a valuable tool for studying the pathogenesis of AAHC. Our aim is to quantify the levels of TM and TV in the gut of mice infected with the wild-type and mutant strains. The results obtained will provide crucial insights into the role played by the transcriptional regulator Lrp in the pathogenesis of AAHC, potentially leading to significant advancements in our understanding of this disease and paving the way for more effective treatments.

This study describes Lrp as a novel positive regulator for the expression and production of TM and TV. Our findings underscore the crucial role of Lrp in the virulence of *K. oxytoca*, raising important implications for further research. The potential therapeutic strategies that could emerge from our research, such as targeting Lrp for drug development or using our findings to guide antibiotic treatments, not only highlight the clinical applications of our discovery in disease management but also inspire hope for the future of treatment. The promising nature of our findings and the potential they hold for developing new therapeutic strategies should encourage and inspire all those researching in translational and clinical fields.
